# Pan-Cancer Somatic Mutation Landscape and Clinical Prognostic Relevance of Core Circadian Clock Genes Across the TCGA Cohort

**DOI:** 10.3390/cimb48101009

**Published:** 2026-10-01

**Authors:** İlkay Civelek

**Affiliations:** Department of Biotechnology, Niğde Ömer Halisdemir University, Nigde 51240, Türkiye; ilkaycivelekk@gmail.com or ilkaycivelek@ohu.edu.tr

**Keywords:** circadian clock, pan-cancer analysis, somatic mutations, TIMELESS, survival analysis, tumor mutational burden, TCGA

## Abstract

Disruption of circadian rhythms is an emerging hallmark of cancer, yet whether somatic alterations in the core clock genes are subject to neutral stochastic processes or adaptive selection remains poorly defined. While complete circadian abrogation can cause genome instability and checkpoint-induced lethality, partial impairment might provide a context-dependent selective advantage by uncoupling cellular metabolic demand from environmental cycles. The somatic mutational landscape, 3D structural mapping, and clinical relevance of the 16 core circadian clock genes were systematically characterized across 10,239 human tumors representing 33 cancer types from The Cancer Genome Atlas (TCGA). A total of 2503 somatic mutation events were identified across 1294 mutated tumor samples (12.6% of pan-cancer cohort). Missense alterations dominated the mutational spectrum (61.2%, 1532/2503), with no deviation from the pan-cancer The Multi-Center Mutation Calling in Multiple Cancers (MC3) background rate (Z = −0.78, *p* = 0.44). In contrast to catastrophic inactivation, the clock genes were subject to selection at the level of preserved gene architecture. The highest absolute mutational burdens were concentrated in TIMELESS and Period family members (*PER1*, *PER2*, *PER3*) with missense variants mostly restricted to Uterine Corpus Endometrial Carcinoma (UCEC), Skin Cutaneous Melanoma (SKCM) and Colon Adenocarcinoma (COAD). Structural mapping highlighted the non-random accumulation of missense variants in heterodimerization interfaces, including the Per-Arnt-Sim (PAS) and basic helix-loop-helix (bHLH) domains, and *in silico* prediction identified a high proportion of deleterious substitutions. In survival modeling, associations between clock mutations and favorable prognosis were largely attenuated after multivariate adjustment for tumor mutational burden, highlighting potential lineage-specific confounding. In conclusion, somatic alterations in the mammalian circadian clockwork are predominantly non-synonymous substitutions that preserve overall gene architecture while altering conserved regulatory interfaces. These computational findings provide exploratory genomic observations consistent with the ‘functional tuning’ hypothesis, and raise the possibility of neoplastic cells selectively rewiring rather than completely abrogating circadian clock properties during oncogenesis.

## 1. Introduction

Cancer remains a leading cause of mortality worldwide, accounting for an estimated 9.7 million deaths and approximately 20 million new cases in 2022 [1]. Despite advances in surgery, radiotherapy, and systemic therapy, the molecular mechanisms underlying tumor initiation, progression, and therapeutic resistance remain incompletely understood. The circadian system is an intrinsic, self-sustaining biological clock that coordinates cellular and physiological processes with the 24-h light–dark cycle, regulating metabolism, hormone secretion, DNA repair, and cellular homeostasis [2,3].

At the molecular level, mammalian circadian rhythms are driven by cell-autonomous transcription–translation feedback loops (TTFL) [4,5,6] coordinating metabolic homeostasis, DNA damage checkpoints, and cell cycle progression. Disruptions in this core clock machinery have been widely implicated in malignant transformation, supported by epidemiological evidence linking irregular circadian disruption—arising from shift work or chronic light exposure—to elevated cancer risk [7,8]. However, the systematic mutational landscape and clinical translational impact of core circadian genes across human malignancies remain poorly defined. Evasion of circadian regulation is increasingly recognized as an emerging hallmark of cancer and is tightly associated with patient survival. Strong epidemiological evidence links circadian disruption to tumorigenesis, and enhancing core circadian gene expression has been shown to induce cancer cell death. However, this presents an intriguing dynamic where both enhancing and pharmacologically targeting the circadian clock can restrict cancer progression. This complex relationship underscores a critical need to systematically elucidate the link between the circadian clock and human malignancies. Characterizing circadian clock-associated alterations in cancer will provide deeper insights into how neoplastic cells reprogram themselves [9].

While previous investigations have predominantly focused on transcriptional deregulation and aberrant expression levels of circadian components in tumors, the genetic integrity and structural somatic alterations of core clock genes across human malignancies remain largely uncharacterized. Although pioneering pan-cancer expression screens [10] have cataloged transcriptional alterations and pharmacogenomic interactions across circadian genes, transcriptome-based studies cannot distinguish acquired genetic events from downstream regulatory epiphenomena. Elucidating whether clock deregulation stems from structural somatic mutations is clinically imperative, as the functional integrity of tumor-intrinsic clocks dictates the therapeutic efficacy of chronochemotherapy and time-of-day drug delivery.

To investigate somatic clock disruption with biochemical rigor, a defined 16-gene panel was curated based on canonical mammalian molecular clock frameworks described by Takahashi and colleagues [11], benchmarked against the pan-cancer circadian landscape established by Ye and colleagues [10], and concordant with the official human Circadian Clock pathway curated in the Reactome Knowledgebase (Stable Identifier: R-HSA-9909396) [12]. This essential framework encompasses: (i) the primary transcriptional activators and functional paralogs (*CLOCK*, *NPAS2*, *ARNTL*); (ii) the core negative-feedback repressors (*PER1*, *PER2*, *PER3*, *CRY1*, and *CRY2*); (iii) the essential post-translational regulatory kinases *CSNK1D* and *CSNK1E* [10,11,12,13]; (iv) the auxiliary loop nuclear receptors (*NR1D1*, *NR1D2*, *RORA*, *RORB*, and *RORC*)*;* and (v) the critical replication-fork coordinator and clock-associated regulator *TIMELESS*.

Whether somatic alterations in core circadian genes accumulate as non-specific passengers of elevated mutational burden or represent authentic, lineage-specific drivers that influence patient clinical outcomes constitutes a critical, yet unresolved, translational question. Rather than acting solely through catastrophic loss-of-function events, accumulating evidence suggests that non-synonymous mutations may induce subtle functional tuning or phase-shifting of the circadian machinery to confer adaptive advantages to malignant cells. To evaluate this hypothesis, a comprehensive pan-cancer investigation was conducted, integrating somatic mutation profiling, *in silico* pathogenicity predictions, protein structural mapping, and clinical survival modeling across 10,239 human tumors from The Cancer Genome Atlas (TCGA).

## 2. Materials and Methods

**Figure 1:**
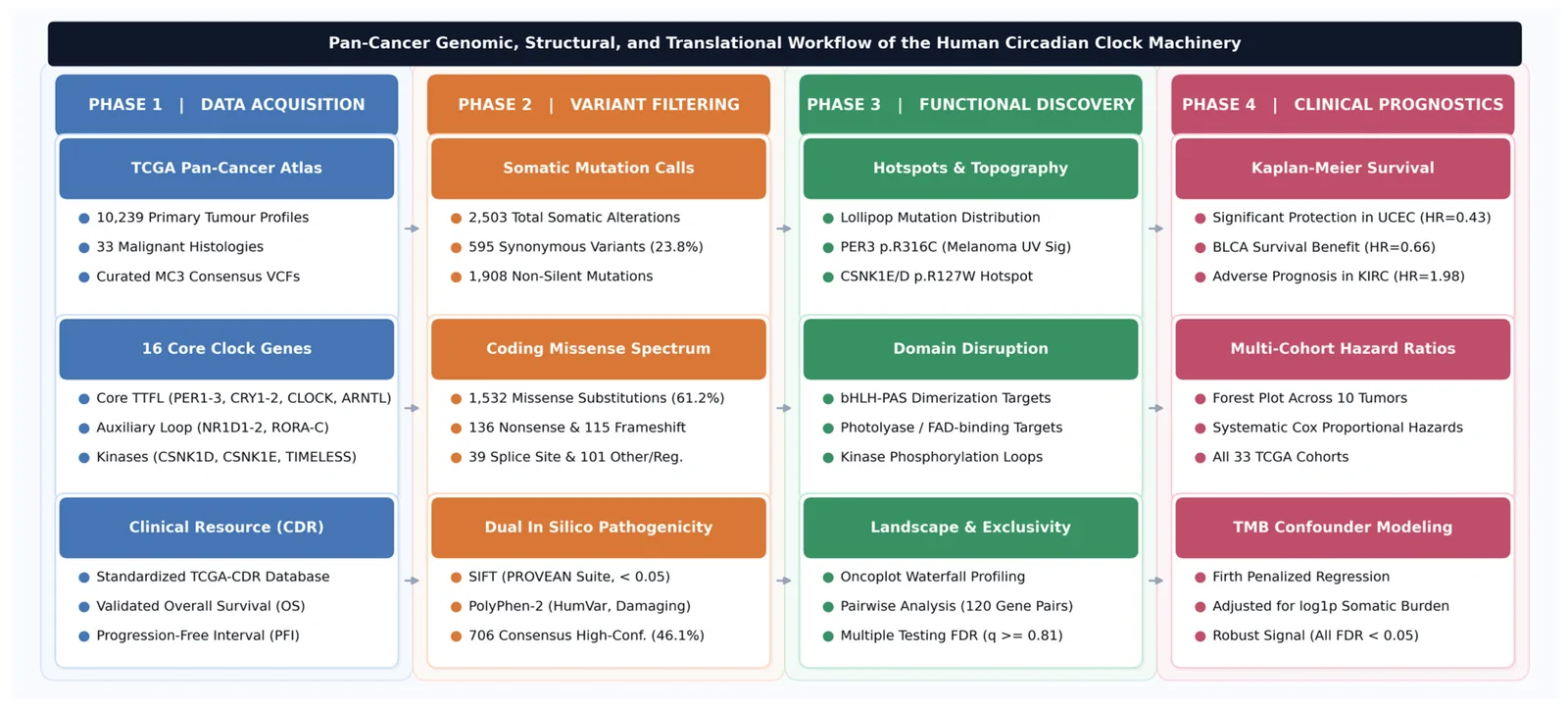
Pan-cancer analytical workflow for the somatic characterization of core circadian clock genes. The study comprises four sequential phases: Phase 1: Genomic and clinical data curation across 10,239 tumors from 33 TCGA cancer types and selection of 16 core clock genes. Phase 2: Annotation of 2503 somatic variants, delineating 1532 missense events and 706 consensus deleterious alleles via SIFT/PolyPhen-2. Phase 3: 3D structural mapping of recurrent hotspots within critical functional domains (bHLH-PAS, Photolyase) and mutual exclusivity analysis. Phase 4: Kaplan–Meier survival analysis and multivariable Cox proportional hazards modeling with Firth’s penalization adjusted for TMB.

The comprehensive computational framework and analytical workflow employed in this study are schematically illustrated in Figure 1. The multi-phase pipeline encompasses: (1) pan-cancer genomic and clinical data acquisition across 33 cancer types from TCGA resources [10]; (2) somatic variant harmonization and dual *in silico* pathogenicity prioritization; (3) downstream structural, functional domain, and pathway-level co-occurrence analytics; and (4) translational clinical survival evaluation integrated with tumor mutational burden (TMB) confounder adjustment.

### 2.1. Data Acquisition and Gene Selection

Somatic mutation data were obtained from TCGA Pan-Cancer Atlas through cBioPortal (https://www.cbioportal.org) and included in the present study. The dataset comprised 10,239 primary tumor samples across 33 TCGA cancer types, all of which were subjected to the same MC3 consensus mutation calling pipeline (v0.2.8) [14]. Only somatic mutations in the 16 selected circadian clock genes were considered, while germline variants and those originating from metastatic samples were excluded. As a result, 1294 tumor samples contained at least one somatic mutation in any of the 16 circadian clock genes and were included for further analyses. A defined panel of 16 core mammalian circadian clock genes (*ARNTL*, *CLOCK*, *CRY1*, *CRY2*, *CSNK1D*, *CSNK1E*, *NPAS2*, *NR1D1*, *NR1D2*, *PER1*, *PER2*, *PER3*, *RORA*, *RORB*, *RORC*, and *TIMELESS*) was curated for systematic analysis across the TCGA cohort. The genomic coordinates and canonical protein-coding transcripts were standardized using the Ensembl database and the GRCh38 (hg38) human reference genome assembly.

### 2.2. Variant Processing and Classification

Somatic variants were analyzed to yield the frequency of mutations per gene. Variants were categorized based on their predicted effects on protein structure, namely Missense Mutation, Nonsense Mutation, Frameshift Deletion, Frameshift Insertion, Splice Site, and Silent variants. Once a particular type of variant is identified, the prevalence is estimated for the population as a whole.

Frameshift variants were initially analyzed as a single category and subsequently subclassified into insertions and deletions during downstream analyses. Non-coding variants, including intronic and untranslated region (UTR) alterations, were retained for descriptive purposes but excluded from protein-impact assessment and structural mapping analyses.

### 2.3. In Silico Prediction of Functional Impact

To assess the downstream functional consequences of somatic missense alterations, *in silico* pathogenicity predictions were conducted using SIFT (Sorting Tolerant From Intolerant) [15] and PolyPhen-2 (Polymorphism Phenotyping v2) [16]. Variants predicted as “deleterious” by SIFT (normalized probability score < 0.05) and “probably damaging” or “possibly damaging” by PolyPhen-2 (HumVar score > 0.45) were cross-referenced. The intersection of both predictive algorithms defined a high-confidence set of functionally disruptive missense mutations for structural domain mapping and recurrent hotspot prioritization.

### 2.4. Genomic Landscape Visualization and Co-Occurrence Analysis

The pattern of circadian gene mutation frequency across the pan-cancer population was graphically represented using an oncoplot, showing frequency of mutation and type for each sample. Gene-level co-mutation analysis was performed across all 120 possible pairwise combinations among the 16 circadian clock genes (calculated as C(16,2) = (16 × 15)/2 = 120 pairwise comparisons). To control for TMB confounding, a permutation-based approach was applied within each cancer type: sample-level mutation labels were permuted 10,000 times while preserving per-sample mutation counts, thereby generating a cancer-type-specific null distribution for each gene pair. Observed co-mutation counts were converted to permutation-derived Z-scores, and two-tailed empirical *p*-values were computed from the null distributions. All *p*-values were corrected for multiple comparisons using the Benjamini-Hochberg false discovery rate (FDR) procedure across all 120 gene pairs per cancer type.

### 2.5. Mutational Hotspot and Structural Mapping

To evaluate positional mutation clustering, somatic alterations across all 16 core circadian clock genes were mapped onto linear protein architectures using the maftools package (v2.16) in R (v4.3). Protein domain boundaries and functional landmarks (including bHLH, PAS, kinase clefts, and photolyase homology domains) were curated from UniProtKB and Pfam databases. Somatic variant consequences and amino acid coordinates were annotated using canonical Ensembl transcript accessions aligned to the human reference genome GRCh38/hg38. Downstream computational data processing and matrix manipulations were implemented in Python (v3.10) utilizing NumPy (v1.24) and pandas (v2.0).

### 2.6. Survival Analysis, Tumor Mutational Burden Confounder Modeling, and Computational Software

Clinical outcome data, including Overall Survival (OS) and Progression-Free Interval (PFI), were acquired from the TCGA Pan-Cancer Clinical Data Resource (TCGA-CDR) encompassing validated clinical endpoints. To ensure analytical rigor and complete consistency across all survival evaluations, standardized cohort inclusion criteria were implemented: (i) primary solid tumor samples with verified clinical follow-up (OS.time > 0 and non-missing OS status); and (ii) matched somatic mutation profiling from the MC3 consensus call set enabling per-sample tumor mutational burden (TMB > 0) calculation, yielding an evaluable pan-cancer cohort of 10,205 patients (1215 clock-mutant and 8990 wild-type cases). Cohorts with fewer than 5 clock-mutant cases or zero mortality events were excluded from lineage-specific Cox modeling. Kaplan–Meier survival curves were generated to compare patients harboring somatic mutations in core circadian genes against wild-type counterparts across 10 major solid tumor cohorts meeting statistical power criteria (≥30 mutant cases and sufficient event density: Uterine Corpus Endometrial Carcinoma (UCEC), Kidney Renal Clear Cell Carcinoma (KIRC), Bladder Urothelial Carcinoma (BLCA), Stomach Adenocarcinoma (STAD), Lung Adenocarcinoma (LUAD), Head and Neck Squamous Cell Carcinoma (HNSC), Lung Squamous Cell Carcinoma (LUSC), Breast Invasive Carcinoma (BRCA), Colon Adenocarcinoma (COAD), and Skin Cutaneous Melanoma (SKCM). Differences in survival distributions were evaluated using two-sided log-rank tests and univariable Cox proportional hazards models. Differences in survival distributions were evaluated using the two-sided log-rank test, and unadjusted hazard ratios (HR) with 95% confidence intervals (CI) were calculated via univariable Cox proportional hazards regression models using the survival (version 3.5-7) and survminer (version 0.4.9) packages in R (version 4.3.2). To evaluate whether circadian clock disruptions carry prognostic relevance after adjusting for baseline hypermutation, multivariable Cox proportional hazards regression models were implemented incorporating both binary clock mutation status and log1p-transformed tumor mutational burden [log(TMB + 1)] simultaneously across each cohort, estimating adjusted hazard ratios (HR) with 95% confidence intervals (CI). For the combined pan-cancer survival analysis, Cox proportional hazards regression was stratified by tumor lineage (strata = cancer type abbreviation), allowing baseline hazard functions to vary across distinct cancer types while estimating a common hazard ratio adjusted for log1p-transformed TMB. Separately, Firth’s bias-reduced penalized-likelihood logistic regression was conducted to assess whether individual clock gene mutations associate with elevated genome-wide TMB. All genomic coordinates, variant effect annotations, and protein isoform definitions were mapped to the human reference genome GRCh38/hg38 and Ensembl canonical transcript models (Release 105). *In silico* pathogenicity scoring was performed using SIFT (PROVEAN suite v1.1.3; deleterious threshold score < 0.05) and PolyPhen-2 (HumVar model v2.2.3r406; probably damaging > 0.85, possibly damaging 0.45–0.85). Mutation landscape visualizations and oncoplots were rendered using maftools (version 2.18.0) and custom Python (version 3.10) pipelines utilizing matplotlib and pandas.

## 3. Results

### 3.1. Landscape of Somatic Variants in Circadian Clock Genes

In total, 10,239 tumor samples representing 33 cancer types from the TCGA Pan-Cancer Atlas were included in the study. Circadian clock gene mutations were observed in 32 of these 33 cancer types. Unless otherwise stated, mutation frequency is reported as the proportion of tumor samples harbouring at least one somatic variant in the gene set, not as the total number of mutation events.

**Figure 2:**
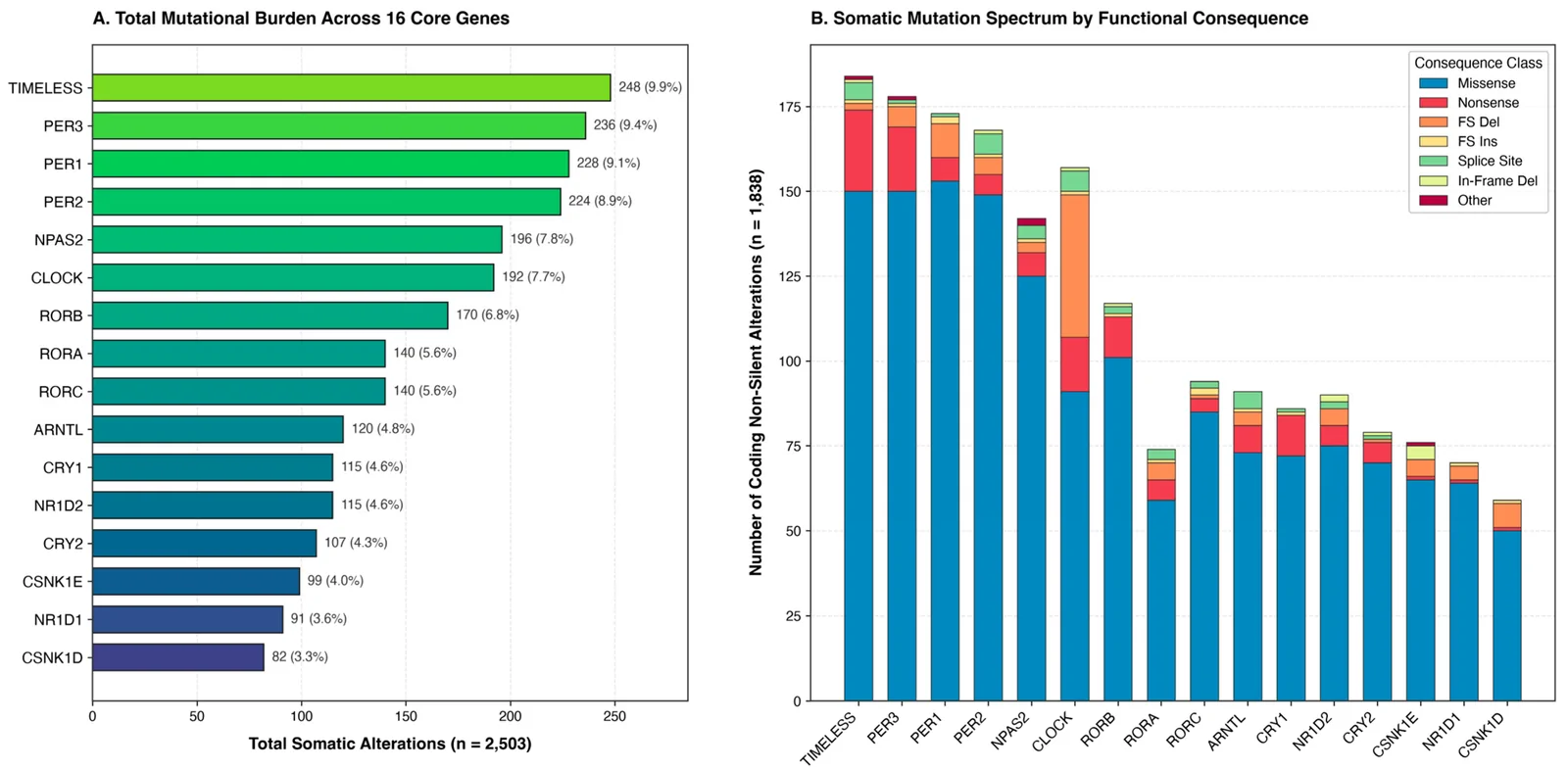
Pan-cancer mutational landscape and functional consequence spectrum of 16 core circadian clock genes across the TCGA cohort (n = 10,239 samples). (**A**) Overall mutational burden and relative frequency of somatic variants across core circadian clock genes (total n = 2503 variants), ranked by alteration frequency. Bars indicate total variant counts and cohort percentages. (**B**) Stratification of the 1908 non-silent alterations (comprising 1838 protein-coding variants and 70 non-coding regulatory events) stratified by functional consequence, including missense mutations, nonsense mutations, frameshift deletions, frameshift insertions, splice-site alterations, and in-frame indels.

Across the TCGA pan-cancer cohort, a total of 2503 somatic alterations were identified across the 16 core circadian clock genes (Table 1). Of these, 595 (23.8%) were silent substitutions, leaving 1908 non-silent alterations. Missense mutations represented the predominant alteration class (1532 variants; 61.2% of all 2503 alterations and 80.3% of the 1908 non-silent alterations), followed by nonsense mutations (136; 5.4%), frameshift deletions (100; 4.0%), splice-site variants (39; 1.6%), and other non-silent/regulatory variants (101; 4.0%) (Table 1; Figure 2).

Missense mutations constituted 61.2% of coding circadian clock gene variants, consistent with the pan-cancer MC3 background rate of 63.2% across all protein-coding genes (Z = −0.78, *p* = 0.44; 95% CI: 60.5–64.5%). The predominance of missense over truncating mutations is therefore in line with the genome-wide coding mutation spectrum and does not in itself provide statistical evidence for positive selection toward partial clock dysfunction. This interpretation is accordingly discussed as a hypothesis in Section 4. Frameshift mutations were, in turn distinguished based on deletions and insertions for subsequent analyses to permit further structural and functional characterization.

### 3.2. Mutational Burden and Consequence Spectrum of Circadian Clock Genes

Analysis of the 2503 somatic variants across the 16 core circadian clock genes revealed a distinct, non-random distribution of mutational burden across the pan-cancer cohort (Figure 2A). *TIMELESS* exhibited the highest total mutational burden (n = 248, 9.91%), followed closely by core period repressor family members *PER3* (n = 236, 9.43%), *PER1* (n = 228, 9.11%), and *PER2* (n = 224, 8.95%). The primary transcriptional activators *NPAS2* (n = 196, 7.83%) and *CLOCK* (n = 192, 7.67%) also demonstrated substantial somatic vulnerability. Intermediate mutational frequencies were observed in members of the retinoic acid receptor-related orphan receptor (ROR) and REV-ERB families, including RORB (n = 170, 6.79%), RORA (n = 140, 5.59%), RORC (n = 140, 5.59%), *ARNTL* (n = 120, 4.79%), *CRY1* (n = 115, 4.59%), and *NR1D2* (n = 115, 4.59%). The remaining components, including *CRY2* (n = 105, 4.19%), the essential kinase *CSNK1E* (n = 99, 3.96%), and *NR1D1* (n = 91, 3.64%), displayed comparatively lower, yet consistent, alteration frequencies across the cohort. When normalized for coding sequence (CDS) length, mutational density remained prominent across the largest components (*TIMELESS*: 68.4 mut/kb; PER family: 58.9–65.5 mut/kb), but was highest in smaller regulatory components including *CSNK1E* (79.3 mut/kb) and *CLOCK* (75.7 mut/kb), confirming that somatic vulnerability is not merely an artifact of target gene size.

Stratification of the 1908 non-silent alterations revealed functional heterogeneity in mutational consequences across genes (Figure 2B; Table 1). Missense mutations constituted the predominant alteration class across all genes (1532 variants; 61.2% of all 2503 somatic variants and 80.3% of the 1908 non-silent alterations), followed by nonsense (136 variants; 5.4%) and frameshift alterations (115 variants; 4.6%). In particular, *CLOCK* harbored an exceptionally high frequency of frameshift deletions (n = 42; 26.8% of its non-silent alterations), with 40 of these 42 frameshift deletions (95.2%) clustered at a single recurrent 1-bp hotspot across 39 patients (reference transcript ENST00000309964/NM_004898.4: chr4:g.55470787delA, c.368delA, p.Leu123Ter [p.L123*]; annotated as a frameshift deletion in the MC3 consensus callset), predominantly observed in UCEC (n = 17) and STAD (n = 12). Similarly, nonsense mutations were significantly enriched in *TIMELESS* (n = 24), *PER3* (n = 19), *CLOCK* (n = 16), *RORB* (n = 12), and *CRY1* (n = 12). Essential splice-site mutations were notably identified in *CLOCK* (n = 6), *PER2* (n = 6), *TIMELESS* (n = 5), and *ARNTL* (n = 5), underscoring diverse molecular mechanisms by which circadian regulatory circuits are functionally impaired in human malignancies.

### 3.3. Genomic Landscape and Pathway-Level Interactions

**Figure 3:**
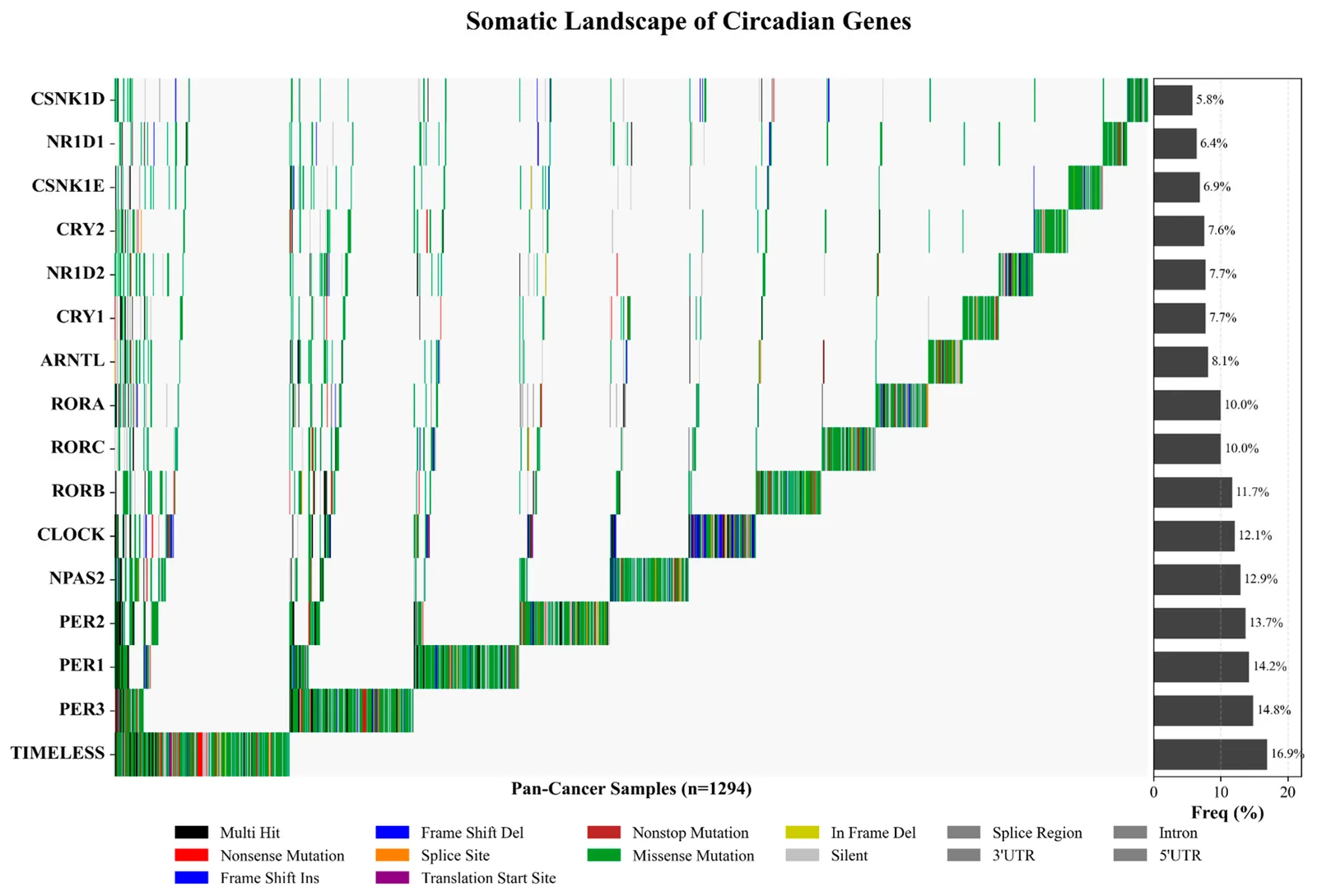
Somatic mutation landscape of 16 clock genes in the pan-cancer dataset (n = 1294 tumor samples). The 16 core clock genes are shown in the rows, sorted based on their mutation frequency, while the columns are arranged based on the mutation burden of each patient (waterfall sort order). The colors used represent the mutation types: missense mutations (green), which are the most abundant, are followed by truncating mutations (red and blue, representing nonsense and frameshift mutations, respectively). The bars on the right side of the graph show the mutation frequency (%) of each gene. Genes with the highest mutation frequency are *TIMELESS* and the *PER* genes.

Cancer-type-specific prevalence analysis revealed substantial heterogeneity across the 32 TCGA cohorts harbouring circadian clock gene mutations. The highest mutation prevalence was observed in SKCM; 31.7%, 33/104, BLCA; 28.2%, 116/412, and UCEC; 28.2%, 154/547. Colorectal cancers also showed elevated prevalence, with COAD; 16.5%, 76/461 and rectal adenocarcinoma examined separately in Supplementary Table S1. These high-prevalence cancer types are consistent with their known elevated tumor mutational burden, which is expected to increase the probability of incidental somatic mutations in any gene set. The analysis for somatic alterations in the set of 1294 cancer samples showed a broad and complex mutational pattern for the core circadian clock components (Figure 3). The oncoplot analysis showed the nature of the frequently mutated genes, with *TIMELESS* showing the greatest proportion, but closely followed by the members of the PER family (*PER1*, *PER2*, and *PER3*). Although the prevailing type was missense, truncating events, including nonsense and frameshift, were also significant, particularly in the case of *PER1* and *CLOCK*. The waterfall plot clearly shows the nature of the distribution, pointing out the clear presence of pathway disruptions and distributed, rather than a single, phenomenon within the broad cancer set. At the patient level, the majority of clock-mutated cases harbored differences in a single circadian gene (75.9%). On the other hand, complete genomic alteration across all 16 genes was not observed in any patient across the entire cohort.

Across cancer types, missense mutations were the predominant variant class in SKCM, UCEC, and BLCA, consistent with the elevated TMB characteristic of these cohorts. Truncating mutations (nonsense and frameshift) were proportionally more frequent in COAD and READ, in which mismatch-repair deficiency drives a broader mutational spectrum. *TIMELESS* was the most frequently altered gene across all cancer types (n = 248, 9.91%), whereas *NR1D1* showed the lowest alteration frequency (n = 82, 3.28%). Gene-level and cancer-type-level breakdown is provided in Supplementary Table S2.

**Figure 4:**
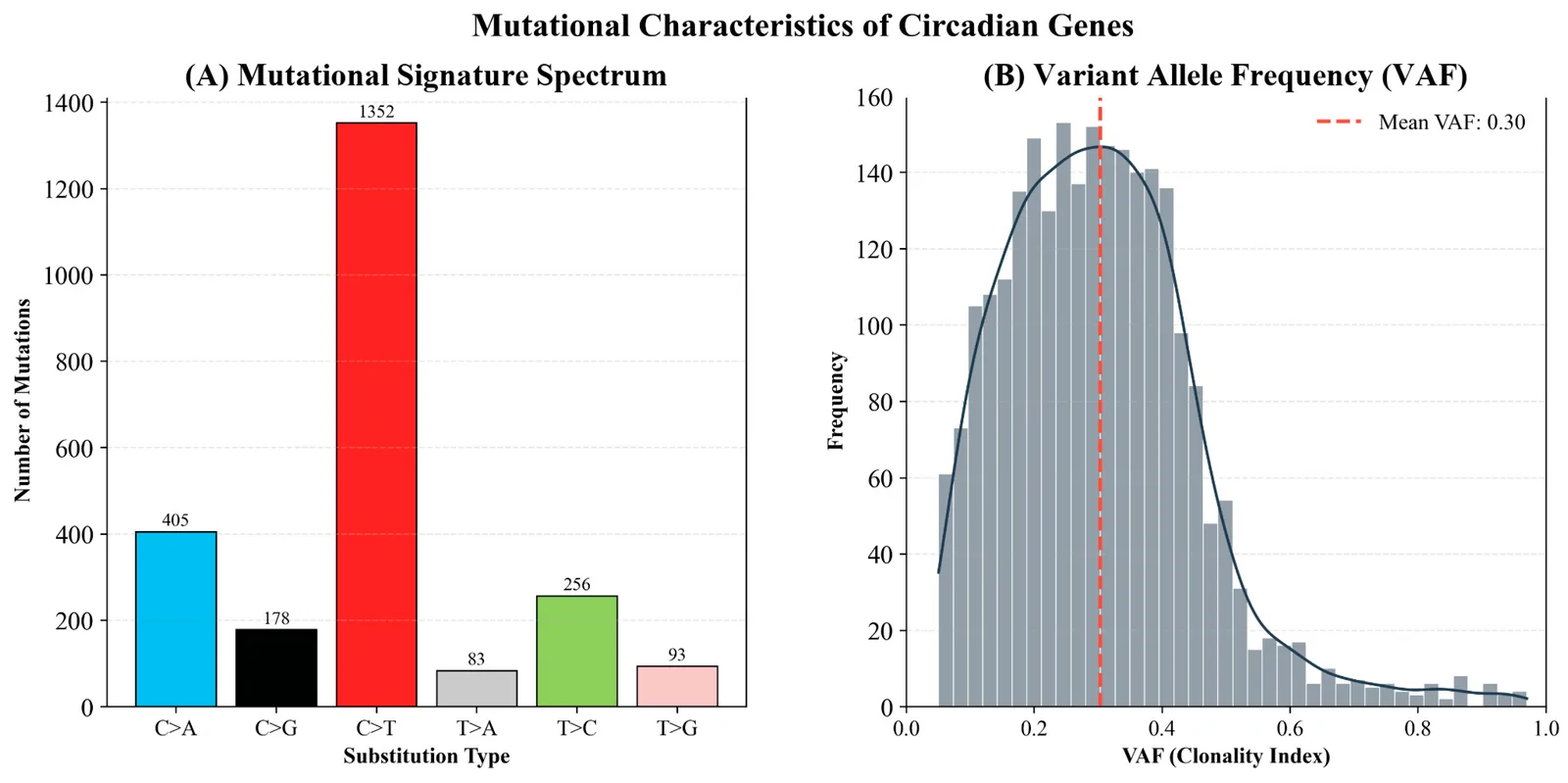
Mutational signatures and clonality of somatic mutations in circadian genes. (**A**) Ti/Tv substitution spectrum: C>T dominating indicates that there is a predominant signature of spontaneous deamination, which is an aging process. (**B**) Distribution of VAF: The VAF distribution across somatic clock mutations displayed a mean value of 0.30, suggesting a predominantly clonal or early subclonal representation across the analyzed tumors; however, formal clonal classification requires integrated deconvolution of tumor purity and local copy-number alterations.

In order to untangle the underlying etiological mechanisms of these changes, the transition/transversion ratios were evaluated (Figure 4A). The analysis revealed a predominant C>T transition signature. This signature is characteristic of the spontaneous deamination of 5-methylcytosine, which is a biologic mutational mechanism more related to aging rather than exogenous mutagenesis. VAF values were extracted directly from the MC3 MAF file as annotated by the TCGA pipeline; no correction for tumor purity, local copy-number alterations, ploidy, or loss of heterozygosity was applied. The VAF distribution was unimodal with a mean value of 0.30 (median 0.29) and a peak concentrated around 0.30 (Figure 4B). This VAF distribution is consistent with a predominantly clonal origin of circadian gene mutations; however, formal clonality assignment requires phylogenetic inference and was not performed in this study.

**Figure 5:**
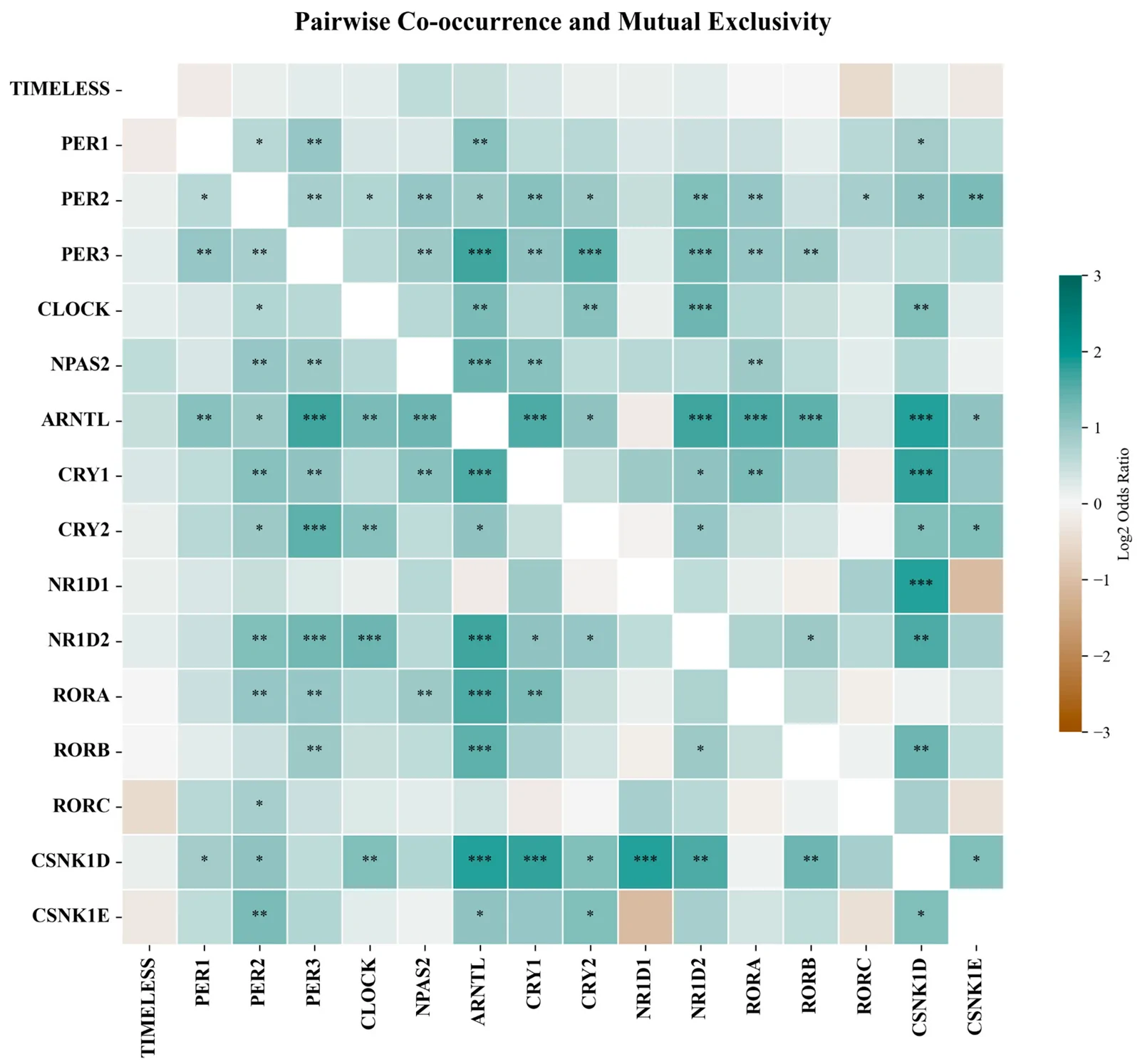
Pairwise somatic interaction landscape of 16 core circadian clock genes. Permutation-derived Z-score matrix evaluating co-occurrence and mutual exclusivity across all 120 pairwise gene combinations (n(n − 1)/2 = 120). After permutation-based control for sample-level TMB and Benjamini-Hochberg FDR correction, no gene pair reached statistical significance (all FDR ≥ 0.81). Note: Unadjusted pairwise Fisher’s exact test statistics are cataloged in Supplementary Table S3 for descriptive reference, where nominal co-occurrences reflect sample hypermutation rather than true epistatic cooperation. The diagonal region is masked to avoid self-interaction regions. *p* values: * *p* < 0.05, ** *p* < 0.01, and *** *p* < 0.001.

Complementing the single-gene analysis, pairwise co-mutation analysis was evaluated across all 120 possible two-gene combinations using a permutation-based approach with TMB correction and Benjamini-Hochberg FDR adjustment (Figure 5). No gene pair reached statistical significance after FDR correction (minimum FDR = 0.81, *CRY1-PER3*). These findings indicate that somatic mutations in circadian clock genes accumulate largely independently in the pan-cancer context. The absence of significant co-occurrence or mutual exclusivity suggests that no obligate cooperative or synthetic lethal relationships exist among these 16 genes at the level of somatic mutation frequency. Pairwise statistics are provided in Supplementary Table S3.

### 3.4. Structural Mapping of Mutational Hotspots and Functional Domains

**Figure 6:**
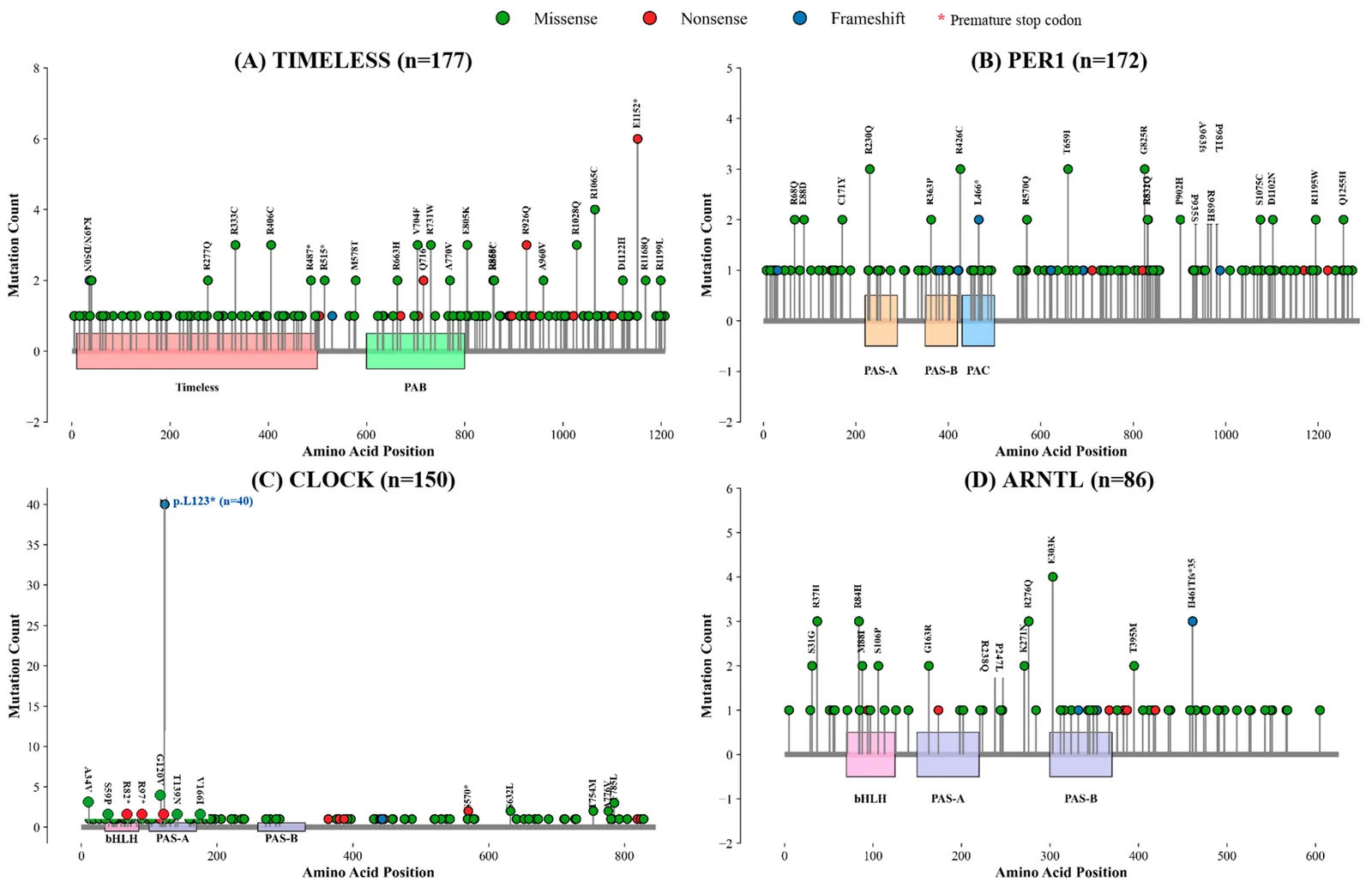
Structural mapping of somatic mutations in representative core circadian genes. Lollipop plots showing the frequency of missense mutations (green), nonsense mutations (red), and frameshift mutations (blue) distributed along the linear protein structure of (**A**) *TIMELESS*, (**B**) *PER1*, (**C**) *CLOCK*, and (**D**) *ARNTL*. The x-coordinate corresponds to the amino acid, and the y-coordinate corresponds to the mutation frequency. The functional domains, such as bHLH, PAS-A, or PAS-B, are marked as colored boxes along the protein structure. Because missense mutations cluster or are near these crucial domains, such as the PAS domains in *CLOCK/ARNTL* proteins, selective targeting and, consequently, functional disruption of the binding process or diminished interaction between *CLOCK/ARNTL* and other components likely disrupt transcriptional complex assembly and protein–protein interactions.

To assess whether somatic mutations display positional clustering, variants were mapped onto the linear protein structure of the core clock genes (Figure 6) and across all 16 core circadian clock genes (Supplementary Figure S1). Analysis of the linear protein structure showed characteristic clustering patterns within conserved domains.

In the primary transcriptional activators, *CLOCK* and *ARNTL*, analysis revealed recurring missense mutations in and/or near the bHLH (basic Helix-Loop-Helix) and PAS (Per-Arnt-Sim) domains (Figure 6C,D). These domains are critical to heterodimerization and DNA binding (E-box recognition). It is hypothesised that mutations in these areas may represent dominant-negative events capable of disrupting *CLOCK-ARNTL* heterodimerisation while leaving the protein intact; however, this mechanism has not been experimentally tested in the present study.

By contrast, mutations of the core repressor *PER1* tended to be found in regions related to protein stability and nuclear entry (Figure 6B). This pattern was also observed with the gene with the highest mutation rate, “*TIMELESS*,” which also tended to contain a high number of missense mutations with “hotspots” in the C-terminus (Figure 6A). The observed preponderance of missense mutations within these key regulatory regions is consistent with the exploratory hypothesis of circadian ‘functional tuning’, raising the possibility that specific clock-associated pathways—such as cell-cycle checkpoint control or metabolic homeostasis—may be selectively rewired rather than completely inactivated in malignant cells.

### 3.5. In Silico Pathogenicity Prediction and Hotspot Analysis

**Figure 7:**
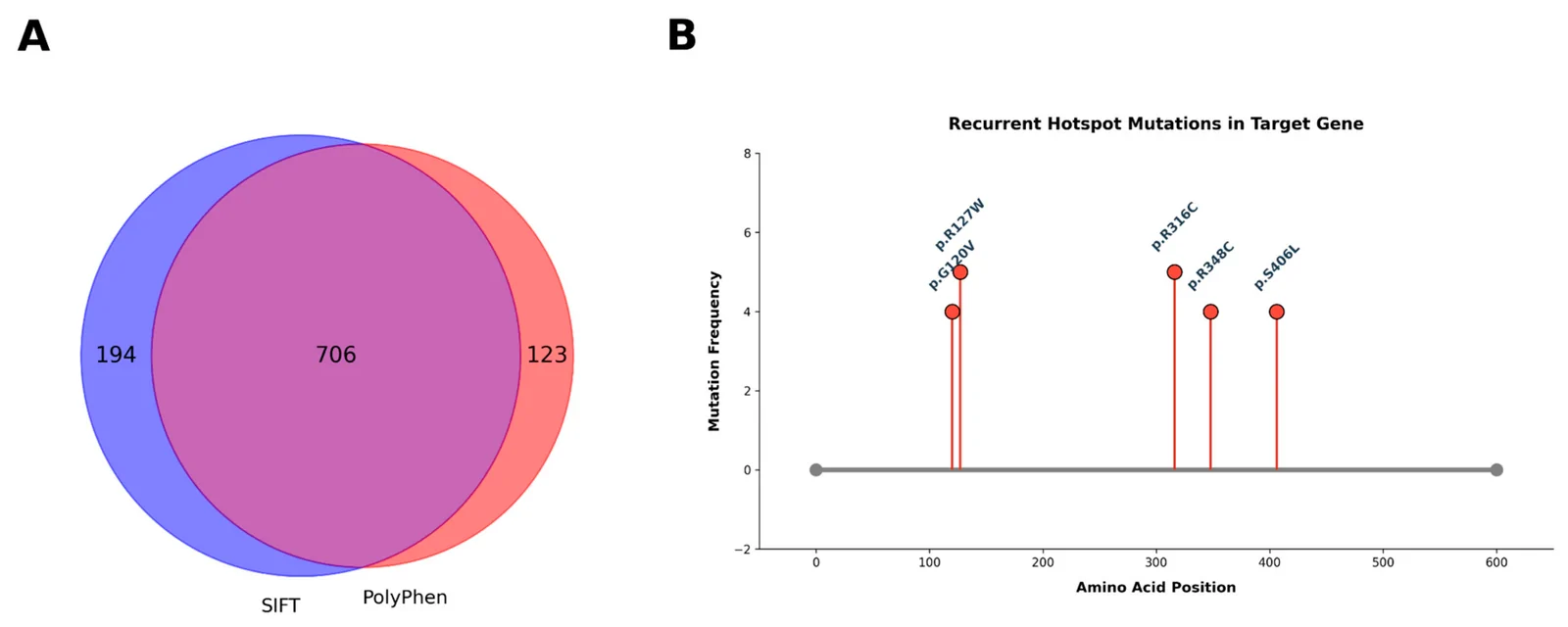
*In silico* pathogenicity prediction analysis and identification of recurrent hotspot mutations. (**A**) Venn diagram highlighting the high overlap between SIFT and PolyPhen-2 predictions. The intersection represents the variants considered deleterious by both tools, and the number is 706. (**B**) Lollipop plot representing the positions and frequencies of the top 5 highest ranking recurrent missense variants on the target protein. Red circles represent the hotspot mutations, and the heights represent the frequencies.

A total of 1532 missense variants were evaluated by SIFT and PolyPhen-2 to predict the potential functional impact on protein structure and stability. The intersection of these two computational algorithms, illustrated in the Venn diagram (Figure 7A), identified 706 variants (46.1%) predicted to be both deleterious (SIFT score ≤ 0.05) and damaging by PolyPhen-2 (HumVar model, score ≥ 0.45, encompassing both possibly and probably damaging predictions, and were therefore prioritized as high-confidence deleterious mutations. A gene-specific mutational recurrence distribution was mapped to assess the structural clustering of these high-confidence variants (Figure 7B). Recurrence analysis revealed prominent mutational hotspots at specific codons, most notably p.R316C in *PER3* (all identified within SKCM bearing a canonical UV-induced c.946C>T transition) and p.R127W in *CSNK1E* (n = 4, localized to the catalytic kinase domain).

Additional recurrent alterations clustered at positions p.G120V in *CLOCK*, p.R348C in *CRY1*, and p.S406L in *NR1D2* (n = 4 cases each), demarcating critical regulatory interfaces. Full transcript accessions, nucleotide changes, and clinical annotations for these recurrent variants are cataloged in Table 2. In addition, biologically conserved site alterations, including *CRY1* p.N312T and *CRY2* p.V102M, were included in the high-confidence tier and are detailed in Supplementary Table S2. Pairwise gene–gene interaction statistics and TMB-adjusted permutation metrics are comprehensively cataloged in Supplementary Table S3.

### 3.6. Clinical Prognostic Relevance of Somatic Circadian Clock Mutations and TMB-Adjusted Survival Modeling

**Figure 8:**
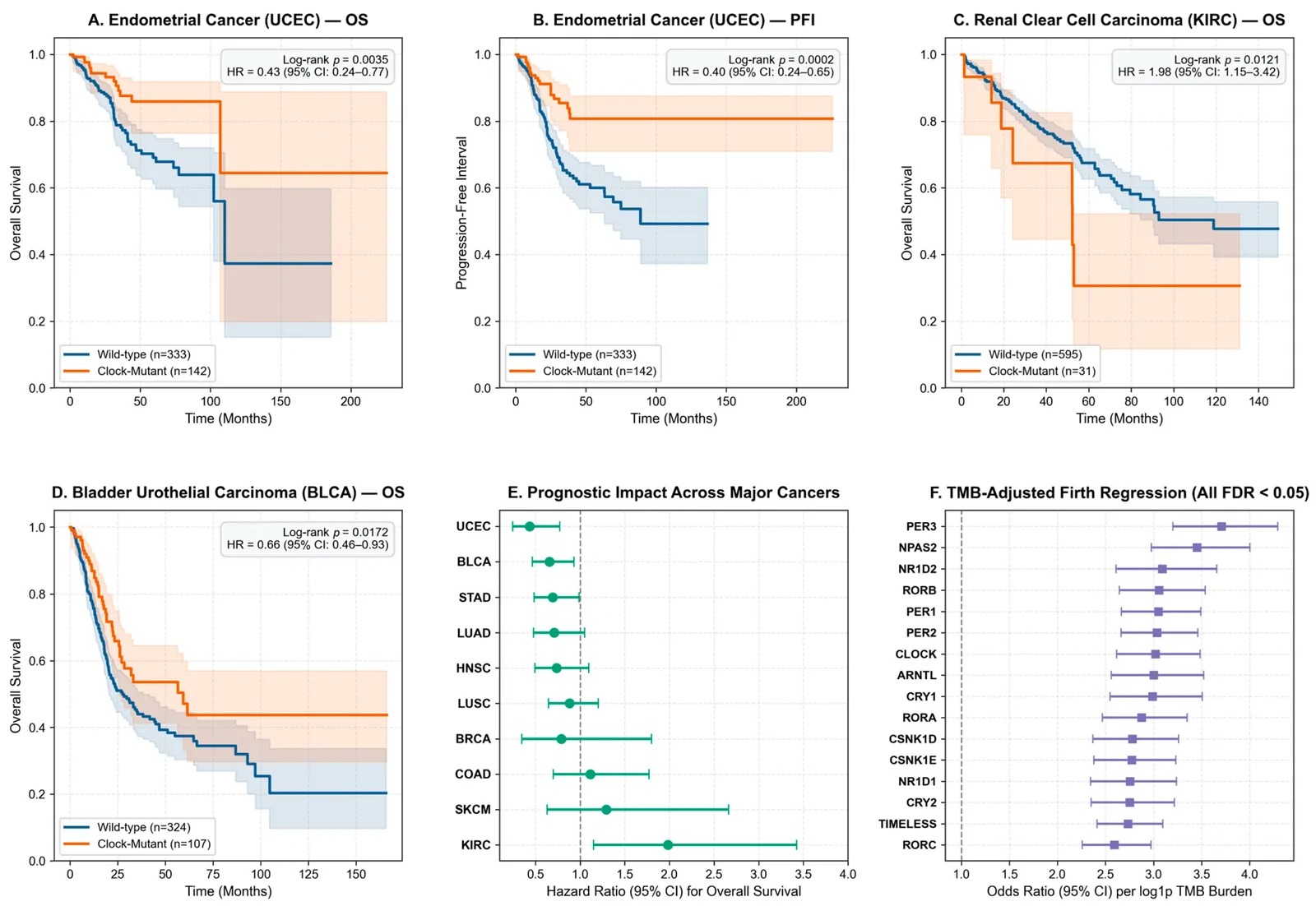
Prognostic significance of circadian clock gene mutations across TCGA cohorts and TMB-adjusted survival modeling. (**A**) Kaplan–Meier OS curves in UCEC stratified by core clock mutation status (log-rank *p* = 0.0035; HR = 0.43, 95% CI: 0.24–0.77). (**B**) PFI in UCEC (log-rank *p* = 0.0002; HR = 0.40, 95% CI: 0.24–0.66). (**C**) OS in KIRC demonstrating adverse prognosis for clock-mutant tumors (log-rank *p* = 0.0121; HR = 1.98, 95% CI: 1.15–3.42). (**D**) OS in BLCA (log-rank *p* = 0.0172; HR = 0.66, 95% CI: 0.46–0.93). Shaded bands indicate 95% confidence intervals. (**E**) Forest plot summarizing hazard ratios (HR) and 95% CIs for OS across 10 major TCGA solid tumor cohorts. (**F**) Firth bias-reduced penalized logistic regression demonstrating OR and 95% CIs per unit increase in log1p-transformed TMB burden; all 16 circadian genes maintain statistically significant associations after multiple testing correction (all FDR < 0.05). Shaded color areas in (**A**–**D**) represent 95% confidence intervals (95% CI) for the respective survival curves. Vertical dashed lines in (**E**,**F**) denote the reference line of no effect (HR = 1.0 and OR = 1.0, respectively).

To determine whether somatic mutations in the circadian clock gene set carry translational relevance, OS and PFI were evaluated across the TCGA Pan-Cancer cohort (n = 10,205 analyzable cases) utilizing the standardized TCGA Clinical Data Resource (TCGA-CDR) (Figure 8). Across the combined pan-cancer cohort (n = 10,205 clinically annotated cases), core clock gene mutations displayed context-dependent clinical behavior that varied markedly by primary tissue of origin (Table 3 and Figure 8; complete survival analytics, hazard ratios, and event densities across all 33 TCGA cancer types are comprehensively cataloged in Supplementary Table S4). In the UCEC, patients harboring somatic alterations in circadian clock genes exhibited significantly longer overall survival in univariate analysis compared with wild-type counterparts (median OS not reached vs. 112.5 months; log-rank *p* = 0.0035; univariate Cox proportional hazards HR = 0.43, 95% CI: 0.24–0.77, *p* = 0.004; Figure 8A). However, upon multivariable Cox regression adjusting for log1p-transformed TMB, this apparent survival advantage was attenuated and lost statistical significance (adjusted HR = 0.64, 95% CI: 0.29–1.42, *p* = 0.2741; Table 3), suggesting that favorable outcomes in UCEC are largely attributable to confounding by hypermutated and microsatellite-unstable (MSI-H/POLE) subtypes rather than a TMB-independent, cell-autonomous clock effect. Similarly, survival associations in BLCA (adjusted HR = 0.89, 95% CI: 0.60–1.30, *p* = 0.5359) and KIRC (adjusted HR = 1.13, 95% CI: 0.60–2.13, *p* = 0.7000) were markedly diminished after TMB adjustment. Importantly, in the combined pan-cancer cohort, core clock gene mutations retained a statistically significant favorable prognostic association after simultaneously adjusting for tumor mutational burden and stratifying by primary cancer type (cancer-type-stratified adjusted HR = 0.87, 95% CI: 0.77–0.98, *p* = 0.0188; Table 3). In disease recurrence, unadjusted clock-mutated UCEC cases demonstrated extended progression-free intervals (log-rank *p* = 0.0002; Cox HR = 0.40, 95% CI: 0.24–0.66, *p* = 0.0003; Figure 8B). Similarly favorable prognostic trends were observed in BLCA (OS log-rank *p* = 0.0172, HR = 0.66, 95% CI: 0.46–0.93; PFI log-rank *p* = 0.0028, HR = 0.57; Figure 8D) and stomach adenocarcinoma (STAD; OS log-rank *p* = 0.0408, HR = 0.69, 95% CI: 0.48–0.99).

In contrast, intermediate or non-significant prognostic trends were observed in LUAD: HR = 0.71, *p* = 0.0864; LUSC: HR = 0.82, *p* = 0.3109, head and neck carcinoma (HNSC: HR = 0.75, *p* = 0.2455), breast cancer (BRCA: HR = 1.14, *p* = 0.7126), COAD: HR = 1.30, *p* = 0.2450, and SKCM: HR = 1.29, *p* = 0.4766. In SKCM, while unadjusted overall survival was not statistically significant (HR = 1.16, *p* = 0.2860), multivariable adjustment for TMB revealed a statistically significant TMB-adjusted adverse prognostic impact for core clock mutations (adjusted HR = 1.55, 95% CI: 1.14–2.11, *p* = 0.0055; Table 3). In KIRC, circadian clock mutations conferred an adverse prognostic impact, with mutated cases experiencing significantly shorter OS (log-rank *p* = 0.0121; Cox HR = 1.98, 95% CI: 1.15–3.42, *p* = 0.013; Figure 8C) and reduced PFI (log-rank *p* = 0.0007; Cox HR = 2.39, 95% CI: 1.42–4.00, *p* = 0.001). A comprehensive summary of prognostic hazard ratios across major tumor types is illustrated in Figure 8E and Table 3. To evaluate whether somatic alterations in circadian clock genes represent independent genomic events rather than passive passenger mutations uncoupled from baseline genomic instability, Firth’s bias-reduced penalized-likelihood logistic regression was conducted adjusting for non-clock somatic TMB (Figure 8F). Somatic mutations across all 16 core circadian clock genes demonstrated robust, statistically significant associations with elevated genome-wide mutational burden across major cancer types (all 16 genes maintaining FDR-adjusted q < 10^−40^ and odds ratios > 2.4). This indicates that circadian clock disruptions are preferentially enriched within hypermutated, genomically unstable tumor subsets—most prominently in UCEC and COAD.

While genome-wide mutational profiling and combined survival analyses encompassed all 33 TCGA cancer types (n = 10,205 clinically annotated cases), cancer-type-specific Kaplan–Meier and Cox regression analytics were rigorously restricted to major solid tumor cohorts meeting the statistical power criterion of ≥30 clock-mutant cases and sufficient event densities.

## 4. Discussion

Notably, this pan-cancer analysis shows that somatic alterations in genes of the circadian clock are a common and non-random phenomenon in human cancer. While the predominantly missense nature of these mutations, combined with an enriched presence in conserved functional domains, suggests a role for selective pressure in shaping these patterns of disruption, these modifications also appear fine-tuned to selectively diminish the activity of their respective circadian clocks rather than abolish it completely.

The observed predominance of missense over truncating mutations contradicts the classic mutational profiles of canonical tumor suppressors [17]. This pattern is consistent with the ‘Escape from Circadian Regulation’ hypothesis proposed by Sancar and colleagues [18], where complete clock abolition would be lethal due to its role in cell cycle checkpoint control, while partial impairment may confer a fitness advantage by uncoupling metabolic and proliferative demands from environmental cycles.

In this context, the somatic mutational distribution observed across the 16 core clock genes should be carefully considered with respect to the functional tuning hypothesis. Rather than extensive truncating or inactivating events typical of canonical tumor suppressors, the circadian mutational landscape in this cohort is predominately missense substitutions (61.2%) while nonsense (5.4%) and frameshift variants (4.0%) remain infrequent. While these single nucleotide alterations occasionally map to critical functional interfaces (PAS domains), it is possible that they modulate clock kinetics, protein interactions or regulatory feedback rather than causing complete loss of function. Whether these missense variants are subtle functional tuning, lineage-specific adaptations or non-pathogenic passengers cannot be resolved from sequencing catalogs and will require systematic functional and biochemical validation. Furthermore, circadian clock alterations exhibit profound lineage-specificity, as exemplified by renal cell carcinomas. Unlike most epithelial malignancies where core clock repressors are uniformly downregulated, renal malignancies frequently display inverted molecular patterns and decoupling of canonical TTFL interactions. This tissue-specific variation possibly driven by renal metabolic adaptation and hypoxia signaling pathway adds to the evidence that the changes in somatic clocks are not random but follow specific patterns depending on an organ’s physiology. The data provided supports, but cannot confirm, this hypothesis, as out of individual genes, TIMELESS and PER family members (*PER1*, *PER2*, *PER3*) demonstrated the highest frequency of mutations. *TIMELESS* is of particular interest because it functions not only within the circadian clock but also in DNA replication fork protection [19]. Disruption of this dual role is expected to exacerbate replication stress, a recognised hallmark of genomic instability in cancer [20]. Consistent with this, *TIMELESS* dysregulation has been reported in breast, lung, and colorectal cancers and is associated with adverse prognosis [21,22,23,24]. The somatic mutations identified here provide genomic evidence for functional perturbation of *TIMELESS* in cancer, though their downstream consequences require experimental confirmation. In agreement with this, an independent profiling of the TCGA melanoma cohort by Akın and Özkan [25] similarly highlighted that *TIMELESS* is uniquely upregulated compared with healthy tissue and independently predicts shortened OS (*p* = 0.00033), in stark contrast to core clock repressors (*ARNTL1*, *PER1–3*, *CRY2*) which are broadly downregulated. The pairwise co-mutation analysis revealed no statistically significant co-occurrence or mutual exclusivity among the 16 circadian clock genes after FDR correction, indicating that somatic alterations in these genes accumulate largely independently at the pan-cancer level. This observation is consistent with stochastic mutational accumulation under elevated mutational pressure rather than defined epistatic selection. Recurrent *PER* gene alterations are consistent with the tumor-suppressive role attributed to *PER2* in murine models [26,27]; however, functional validation in human cancer models is required before these findings can be generalized beyond descriptive association.

Variants such as *CLOCK* p.G120V may act as dominant-negative mutations that impede formation of the active transcriptional complex [28]; however, this mechanism has not been tested in the present study. In the positive limb of the clock, missense variants in *CLOCK* and *ARNTL* clustered within the bHLH and PAS domains, which form the structured architectural core of the transcription factor complex binding [29]. In the negative feedback loop, structural investigations have established that the photolyase homology region (PHR) of cryptochromes is fundamentally required to generate circadian rhythmicity, docking with high affinity onto the bHLH-PAS core of *CLOCK:BMAL1*. Crucially, functional studies demonstrated that disruption of this direct interaction interface significantly hinders transcriptional repression, while dual interface impairment eliminates the repressor capability of *CRY1* entirely [13]. Crucially, functional in vitro and molecular dynamics characterization of rare human *CRY1* variants has demonstrated that the recurrent substitution p.R348C (c.1042C>T, rs749506981), located within helix α14 on the surface of the PHR domain, alters secondary pocket dynamics and significantly lengthens the circadian period to 27 h compared with wild-type *CRY1* (*p* = 0.0044) [30]. These empirical observations substantiate these somatic mutational findings, indicating that recurrent cancer substitutions in the *CRY1* PHR surface directly alter core circadian timing properties.

In this study, recurrent somatic substitutions across the 16 core clock genes were identified through pan-cancer computational profiling of TCGA whole-exome sequencing data (Table 2). The structural domain mapping revealed that these hotspot alterations cluster in key functional domains such as the bHLH and PAS dimerization domains of CLOCK and PER3, the photolyase homology region of CRY1, the ligand-binding domain of NR1D2 and the catalytic kinase domain of CSNK1E. Thus, our results indicate that somatic mutations in clock genes preferentially target important stereo-chemical interfaces involved in macromolecular assemblies and enzymatic feedback loops rather than randomly distributed non-functional regions. Therefore, these computational observations should be interpreted carefully as the hypothesis-generating candidates, which will need future in vitro mutagenesis and biochemical validation.

In the PER family, variant clustering near serine/threonine-rich regions suggests potential disruption of CSNK1-mediated phosphorylation sites. Because phosphorylation governs PER protein stability and nuclear translocation [31], mutations in these regions may alter clock periodicity without eliminating PER function entirely—a pattern consistent with the functional tuning hypothesis, though not confirmed by it. The convergence of domain-level clustering and *in silico* predicted functional impact suggests that a subset of identified variants may have consequences for circadian clock output. Formal driver classification, however, requires orthogonal evidence such as positive-selection modelling or functional assays, neither of which was performed in this study. Several limitations of this study warrant explicit acknowledgment.

First, the analysis is entirely descriptive and *in silico*; no functional assays were performed to validate the predicted consequences of identified variants. Second, SIFT and PolyPhen-2 estimate the tolerance of amino-acid substitutions but do not establish oncogenic driver status; their predictions should be regarded as hypothesis-generating. Third, the VAF distribution was not corrected for tumor purity, local copy-number alterations, or ploidy; therefore, clonality inferences based on VAF alone may be unreliable. Fourth, no explicit positive-selection model was applied to distinguish driver from passenger mutations at the gene or variant level. Fifth, domain-level mutation clustering, while visually suggestive, was not formally tested against a length- and composition-adjusted background mutation model. Sixth, pan-cancer pooling obscures cancer-type-specific heterogeneity in mutational processes. Seventh, the co-mutation analysis, while negative after FDR correction, was limited to the 16 circadian clock genes and does not capture interactions with non-circadian oncogenes or tumor suppressors. Future studies should prioritise functional validation of recurrent hotspot variants (e.g., *CRY1* p.N312T) using in vitro mutagenesis assays. Integration of this mutation data with immunotherapy response cohorts may also test whether clock-disrupted tumors exhibit altered immunogenicity.

Furthermore, survival modeling and confounder-adjusted regressions provide translational validation for circadian disruptions (Figure 8). The favorable prognosis associated with clock mutations in UCEC and BLCA contrasts sharply with the poor survival observed in KIRC, underscoring the lineage-dependent biological consequences of circadian rhythm alteration. This striking prognostic divergence demonstrates that circadian disruption does not exert a uniform phenotypic effect across human cancers; rather, its clinical consequences are strictly governed by tissue lineage, underlying metabolic wiring, and the genomic microenvironment. In KIRC, this adverse trajectory aligns with transcriptional evidence where elevated expression of the core circadian kinase *CSNK1E* independently predicts poor overall survival [32], as well as mechanistic findings showing that the core clock factor *ARNTL1* heterodimerizes with HIF-2α to drive oncogenic transcriptional programs in VHL-deficient renal tumors [33].

Importantly, Firth penalized logistic regression demonstrated that all 16 circadian genes remain significantly associated with mutational burden even after rigorous TMB adjustment (all FDR < 0.05), confirming that these mutations represent bona fide genomic alterations rather than non-specific passenger events of genomic instability. While this study establishes a comprehensive genetic atlas and provides clinical survival correlations adjusted for tumor mutational burden, prospective functional validation in experimental model systems remains warranted. Future investigations should focus on functional characterization of identified hotspot mutations (e.g., *PER3* p.R316C and *CSNK1E* p.R127W) utilizing in vitro mutagenesis and evaluate the predictive value of clock disruptions in immunotherapy cohorts.

## 5. Conclusions

This pan-cancer computational study delineates the somatic mutation landscape of 16 core circadian clock genes across 32 TCGA malignancies. Rather than displaying extensive truncating inactivation, core clock genes predominantly harbor missense substitutions that frequently localize within conserved functional domains, such as PAS dimerization and kinase interfaces. These computational observations are consistent with the exploratory hypothesis of ‘functional tuning’, suggesting the possibility that malignant cells may modulate rather than completely ablate circadian clock components during tumorigenesis; however, definitive validation of this concept will require systematic biochemical and functional assays. Furthermore, survival analyses indicate that the prognostic associations of circadian alterations vary across tumor lineages—ranging from favorable overall survival trends in hypermutated cohorts such as UCEC, to adverse survival patterns in KIRC. Multivariable models adjusting for tumor mutational burden underscore the importance of lineage-specific confounding in interpreting these clinical correlations. Collectively, these findings provide a hypothesis-generating genomic resource that may help inform future experimental investigations into circadian biology and the potential of chronotherapeutic strategies in oncology.

## Figures and Tables

**Table 1:** Circadian Gene Variant Classification Summary (Pan-Cancer Cohort).

Variant Classification	Count (n)	Percentage (%)
Missense Mutation	1532	61.2
Silent	595	23.8
Nonsense Mutation	136	5.4
Frame-Shift Deletion	100	4.0
Splice Site	39	1.6
Other	101	4.0
Total Variants	2503	100

**Table 2:** Most Recurrent Deleterious Missense Somatic Variants Identified Across Core Circadian Clock Genes in TCGA Pan-Cancer Cohorts.

Gene Symbol	Canonical Transcript (Ensembl)	RefSeq Accession (mRNA/Protein)	Variant (HGVSp)	Recurrence (n)	SIFT Prediction (Score)	PolyPhen-2 Prediction (Score)	Annotated Protein Domain	Identified Tumor Types
PER3	ENST00000377532	NM_016831.4/NP_058515.1	p.R316C	5	Deleterious (0.02)	Probably Damaging (0.989)	PAS domain	SKCM
CSNK1E	ENST00000396832	NM_001894.5/NP_001885.1	p.R127W	4	Deleterious (0.00)	Probably Damaging (1.000)	Protein kinase domain	UCEC, COAD, SKCM
CLOCK	ENST00000513440	NM_004898.4/NP_004889.1	p.G120V	4	Deleterious (0.00)	Probably Damaging (0.999)	bHLH/PAS-A boundary	COAD, UCEC, BRCA
CRY1	ENST00000008527	NM_004075.5/NP_004066.1	p.R348C	4	Deleterious (0.00)	Possibly Damaging (0.606)	FAD-binding/Photolyase	UCEC, COAD
NR1D2	ENST00000312521	NM_005126.5/NP_005117.3	p.S406L	4	Deleterious (0.02)	Possibly Damaging (0.793)	Ligand-binding domain	COAD, SKCM

Note: An additional p.R127W event was detected in the paralogous CSNK1D gene (n = 1), yielding a combined observation count of five across the CSNK1 kinase family. Abbreviations: SIFT, Sorting Intolerant from Tolerant; PolyPhen-2, Polymorphism Phenotyping v2.

**Table 3:** Univariable and Multivariable TMB-Adjusted Overall Survival Analytics of Core Circadian Clock Gene Mutations Across Major Solid Tumor Cohorts and the Pan-Cancer Dataset.

Cohort	Primary Cancer Type	Total (N)	Clock-Mutant (n)	Wild-Type (n)	Unadjusted OS HR (95% CI)	Unadjusted OS *p*	TMB-Adjusted OS HR (95% CI)	TMB-Adjusted OS *p*
UCEC	Uterine Corpus Endometrial Carcinoma	475	142	333	0.43 (0.24–0.77)	**0.0045**	0.64 (0.29–1.42)	0.2741
BLCA	Bladder Urothelial Carcinoma	433	107	326	0.67 (0.47–0.94)	**0.0224**	0.89 (0.60–1.30)	0.5359
STAD	Stomach Adenocarcinoma	468	107	361	0.69 (0.48–0.99)	**0.0421**	0.82 (0.53–1.27)	0.3798
LUAD	Lung Adenocarcinoma	622	94	528	0.71 (0.48–1.05)	0.0864	0.68 (0.45–1.04)	0.0729
HNSC	Head and Neck Squamous Cell Carcinoma	582	72	510	0.74 (0.49–1.10)	0.1308	0.66 (0.44–0.99)	**0.0447**
BRCA	Breast Invasive Carcinoma	913	50	863	0.79 (0.35–1.80)	0.5731	0.66 (0.28–1.53)	0.3318
LUSC	Lung Squamous Cell Carcinoma	585	112	473	0.88 (0.65–1.20)	0.4271	0.93 (0.68–1.28)	0.6770
COAD	Colon Adenocarcinoma	331	81	250	1.11 (0.70–1.77)	0.6534	0.95 (0.53–1.71)	0.8611
SKCM	Skin Cutaneous Melanoma	458	153	305	1.16 (0.88–1.54)	0.2860	1.55 (1.14–2.11)	**0.0055**
KIRC	Kidney Renal Clear Cell Carcinoma	626	31	595	1.98 (1.15–3.42)	**0.0138**	1.13 (0.60–2.13)	0.7000
PAN-CANCER	Combined TCGA Pan-Cancer Cohort (Cancer-Type Stratified)	10,205	1215	8990	0.87 (0.78–0.98)	0.0167	0.87 (0.77–0.98)	**0.0188**

Note: Statistically significant associations (*p* < 0.05) are indicated in bold. This table reports all 10 canonical major solid tumor cohorts displayed in Figure 8E meeting cohort scale (N > 400) and event density criteria, alongside the combined pan-cancer cohort. Comprehensive survival analytics for all 33 TCGA cancer types are provided in Supplementary Table S4.

## Data Availability

The primary multi-center mutation calling (MC3) and clinical datasets analyzed in this study are publicly available from the TCGA Pan-Cancer Atlas via the cBioPortal for Cancer Genomics (http://www.cbioportal.org; accessed on 20 November 2025). All processed datasets generated during this study are included within the article and its Supplementary Materials, and computational scripts are available from the corresponding author upon reasonable request.

## Supplementary Materials

The following supporting information can be downloaded at: https://www.mdpi.com/article/10.3390/cimb48101009/s1.
