# Supplementary material for: Pan-Cancer Somatic Mutation Landscape and Clinical Prognostic Relevance of Core Circadian Clock Genes Across the TCGA Cohort
Source: Curr Issues Mol Biol. 2026 Oct 1;48(10):1009. doi: 10.3390/cimb48101009 (PMC13702863; doi:10.3390/cimb48101009)
Supplement: Supplementary file 1 [file cimb-48-01009-s001.zip › cimb-4565448-Supplementary REVISED.pdf]

Supplementary Materials

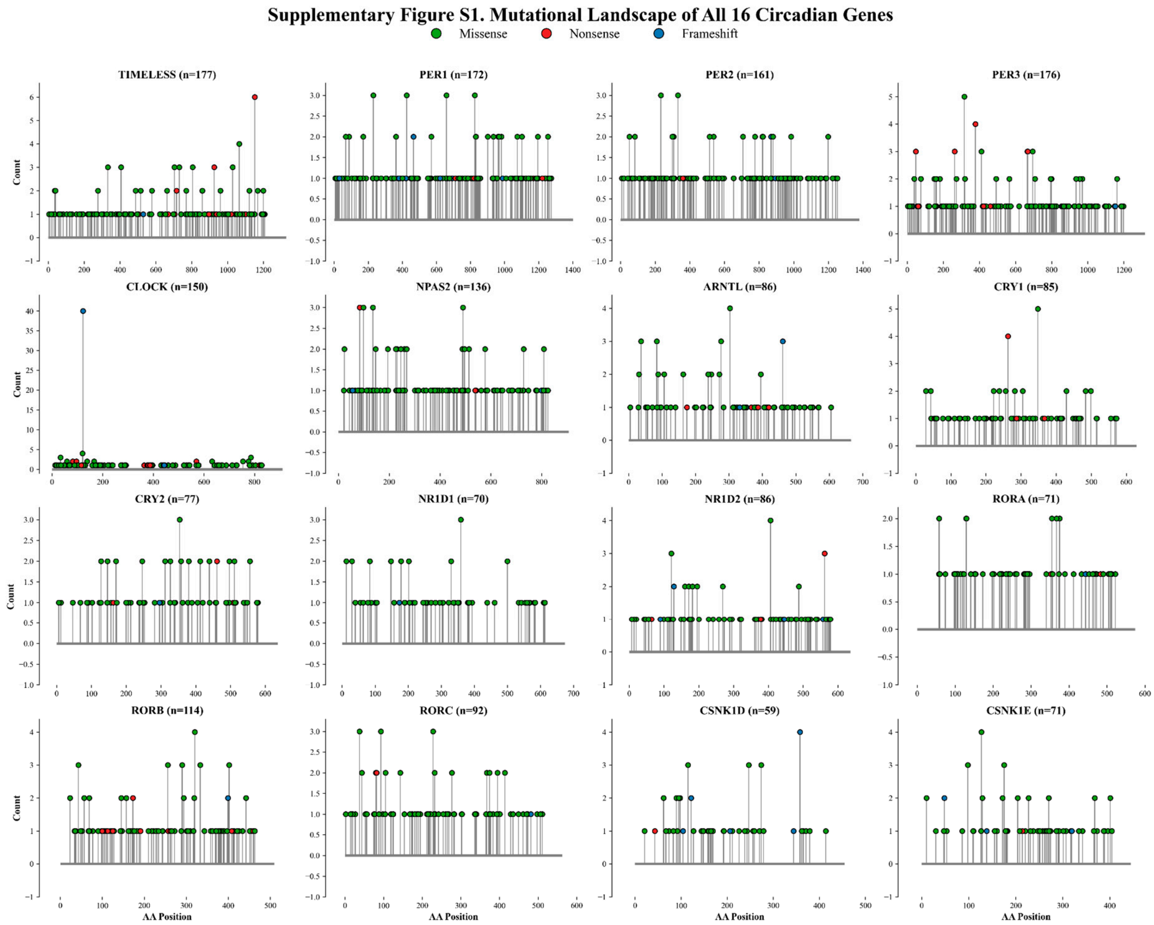

Supplementary Figure S1. Comprehensive mutational landscapes across all 16 core circadian clock genes. High-resolution multi-panel lollipop representations depicting the positional distribution, protein domain localization, and variant classifications across the entire primaryTTFL network (ARNTL, CLOCK, NPAS2, CRY1, CRY2, PER1, PER2, PER3), accessory regulatory feedback loop (NR1D1, NR1D2, RORA, RORB, RORC), casein kinase regulators (CSNK1D, CSNK1E), and TIMELESS. Colored markers denote functional classes (green: missense mutations; black: nonsense/truncating; red: frameshift indels; purple: splice-site).

Supplementary Table S1. Comprehensive catalog of 1,532 somatic missense variants and in silico pathogenicity predictions across 16 circadian clock genes. Full dataset available in "Supplementary Table S1.xlsx".

Supplementary Table S2. Functional domain localization and pathogenicity scores of recurrent hotspot and critical regulatory site variants. Full tabular dataset available in "Supplementary Table S2.xlsx".

Supplementary Table S3. Pairwise statistical analysis of gene-gene co-occurrence and mutual exclusivity patterns across 16 circadian genes. Full dataset available in "Supplementary Table S3.xlsx".

Supplementary Table S4. Pan-cancer overall survival and clinical prognostic analytics of core circadian clock gene mutations across all 33 TCGA cohorts. Full dataset available in "Supplementary Table S4.xlsx".
